# Estimation of carbon dioxide emissions from the megafires of Australia in 2019–2020

**DOI:** 10.1038/s41598-021-87721-x

**Published:** 2021-04-15

**Authors:** Tomohiro Shiraishi, Ryuichi Hirata

**Affiliations:** Center for Global Environmental Research, Earth System Division, National Institute for Environmental Studies (NIES), 16-2 Onogawa, Tsukuba, Ibaraki 305-8506 Japan

**Keywords:** Environmental sciences, Natural hazards

## Abstract

Catastrophic fires occurred in Australia between 2019 and 2020. These fires burned vast areas and caused extensive damage to the environment and wildlife. In this study, we estimated the carbon dioxide (CO_2_) emissions from these fires using a bottom-up method involving the improved burnt area approach and up-to-date remote sensing datasets to create monthly time series distribution maps for Australia from January 2019 to February 2020. The highest monthly CO_2_ emissions in Australia since 2001 were recorded in December 2019. The estimated annual CO_2_ emissions from March 2019 to February 2020 in Australia were 806 ± 69.7 Tg CO_2_ year^−1^, equivalent to 1.5 times its total greenhouse gas emissions (CO_2_ equivalent) in 2017. New South Wales (NSW) emitted 181 ± 10.2 Tg CO_2_ month^−1^ in December 2019 alone, representing 64% of the average annual emissions of Australia from 2001–2018. The negative correlation observed between CO_2_ emissions and precipitation for 2001–2020 was 0.51 for Australia. Lower than average precipitation and fires in high biomass density areas caused significant CO_2_ emissions. This study helps to better assess the performance of climate models as a case study of one of the major events caused by climate.

## Introduction

Catastrophic fires pose risks to humans in Australia for many millennia^1^. Previous catastrophic fires include Black Friday in 1939, Ash Wednesday in 1983, and Black Saturday in 2009. Extensive fires again affected Australia in the 2019 to 2020 season. The burnt area from these fires exceeded that of the 1939 Black Friday fires, which burnt approximately two million hectares of temperate forests, the largest land area since European settlement^2^. The fires began before spring in June 2019, then significantly worsened in early November 2019. Although the fires were dampened by heavy rains in the middle of January 2020, they then re-emerged in early February due to rising temperatures, droughts, and strong winds. Finally, the fires were contained by severe rainstorms in the middle of February^3^. Approximately three million hectares were burnt in the eastern states of Queensland and New South Wales (NSW)^4^. Nolan et al.^2^ reported that these fires burnt 3.8 million hectares of mainly temperate forest in the state of NSW, and 0.5 million hectares in Victoria. Boer et al.^5^ reported that approximately 5.8 million hectares of mainly temperate broadleaf forest were burnt in NSW and Victoria between September 2019 and early January 2020. According to the Center for Disaster Philanthropy (CDP) website^3^, the fires affected not only the landscape, but also humans and animals. At least 34 people, including 25 in NSW, one in Australian Capital Territory, five in Victoria, and three in South Australia (SA) have died since October 2019 due to the wildfires. Furthermore, approximately 3,000 houses and thousands of buildings were destroyed by the fires nationwide. The fires also affected ecosystems; over a third of the koala population has been estimated to have been killed, and hundreds of thousands of fish died in the Macleay River in northern NSW because of the ash and sludge from the fires. The CDP explained some of the causes of these fires as follows. Australia experienced its hottest year on record in 2019, with average temperatures 1.52 °C above the 1961–1990 average. The same year, 2019, turned out to be its driest year with rainfall 40% lower than average based on records going as far back as 1900.

Biomass burning occurs in all vegetated terrestrial ecosystems and strongly affects global carbon cycles by releasing a massive amount of carbon dioxide (CO_2_) into the atmosphere^6–9^. The largest source of global carbon emissions, excluding fossil fuel emissions, is fires—mainly in grasslands and savannas^8^. Vegetation in Africa and Australia significantly contribute to the global emission budget^10^. The Australian government established the Emissions Reduction Fund in 2014 to store carbon or reduce greenhouse gas (GHG) emissions^11^. Extremely severe fires had been relatively rare in southeast Australia, due to strategic fuel management conducted by the government^12,13^. Despite these efforts, massive fires broke out in 2019–2020 season and emitted significant amounts of CO_2_ into the atmosphere. It is important to understand the causes of these large-scale fires and their impact on the climate, ecosystems, society, and economy.

Pickrell^4^ and Nolan et al.^2^ documented the extent of the land areas affected by these fires up to December 2019. The fires continued in NSW and Victoria until February 2020^3^, and CO_2_ emissions for January and February 2020 have not been quantified. Therefore, we estimated the CO_2_ emitted by the Australian fires until February 2020 and created monthly CO_2_ emission maps from these fires to understand changes in the time series and distribution of the fires across the whole of Australia. This paper covers the following: (1) providing CO_2_ emissions and its spatio-temporal distribution; (2) quantitation of the effect on the CO_2_ emission estimation by input sources; (3) evaluation of the relationships between CO_2_ emissions and precipitation and CO_2_ emissions and temperature. It is important to evaluate many case studies of major events to understand the global environment. As an assessment of one of the major events, this study helps to better assess the performance of climate and fire models.

## Results

**Figure 1:**
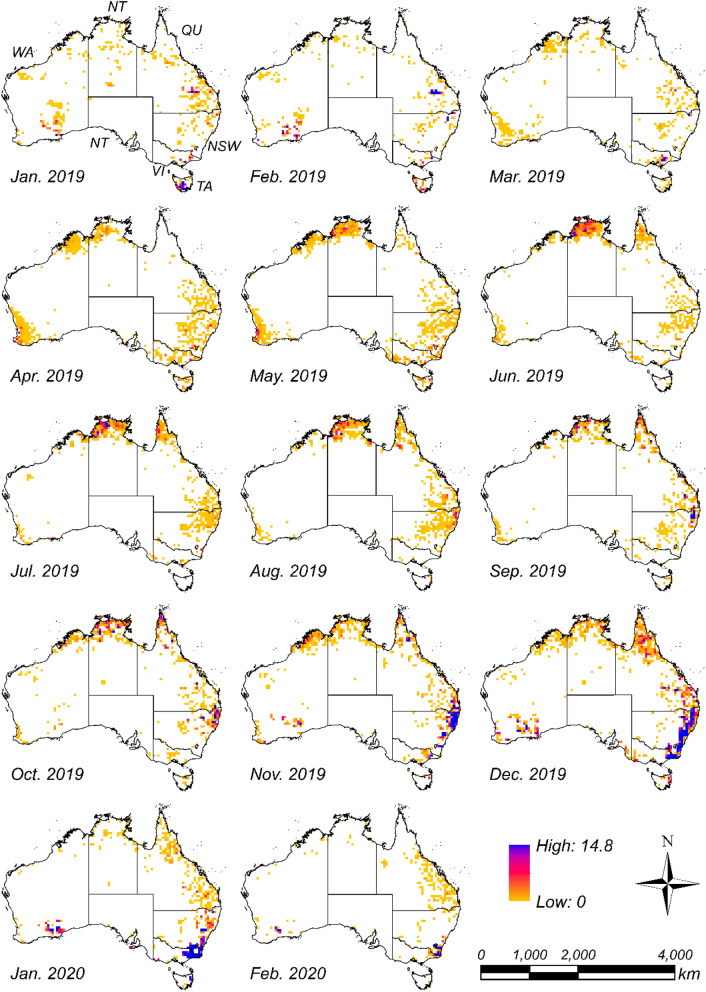
Estimated monthly CO_2_ emissions (Tg CO_2_ grid^−1^ month^−1^) time series between January 2019 and February 2020. CO_2_ emissions are the total amount in each 50 km × 50 km grid. Abbreviations in the top-left figure for January 2019 indicate the state names, namely, New South Wales (NSW), Queensland (QU), South Australia (SA), Tasmania (TA), Victoria (VI), Western Australia (WA), and Northern Territory (NT). Maps were created with ArcGIS version 10.5 (https://www.arcgis.com/).

CO_2_ emissions from fires were estimated for six states and one territory (Fig. S1). The spatio-temporal distribution of estimated monthly CO_2_ emissions between January 2019 and February 2020 are shown in Fig. 1. CO_2_ emissions, which began in northern Western Australia (WA) in March 2019, passed through Northern Territory (NT) and reached Cape York Peninsula in Queensland in June 2019. While continuing to emit CO_2_ in the northern area of the country, the CO_2_ emissions increased in the eastern parts of both Queensland and NSW from April 2019. Afterwards, CO_2_ emissions were estimated in Queensland and NSW from November to December 2019, and in Victoria in January 2020. Other CO_2_ emissions were also noted in southwestern WA from April to May 2019. Although CO_2_ emissions were estimated in Queensland in February 2020, emissions had disappeared in most of the regions by this time (Fig. 1).

Annual CO_2_ emissions from fires in Australia for 2019 were 674 ± 57.6 Tg CO_2_ year^−1^, which was estimated to be 2.4 times the average annual CO_2_ emissions in 2001–2018 (Table 1). Focusing on the monthly CO_2_ emissions, the average emissions from April to October 2001–2018 were consistently large and the emissions in 2019–2020 season increased again after October. In particular, the emissions in November, December 2019, and January 2020 were 157 ± 23.1, 304 ± 16.9, and 173 ± 6.14 Tg CO_2_ month^−1^, respectively. These emissions correspond to 3.6, 9.7, and 6.4 times the average monthly CO_2_ emissions in 2001–2018, respectively.

**Table 1:** Average and one standard deviation of monthly CO_2_ emissions (Tg CO_2_ month^−1^) between 2001–2018 and 2019–2020 in Australia.

Month	Monthly CO_2_ emissions (Tg CO_2_ month^–1^)		
	2001–2018	2019	2020
Jan	27.1 ± 10.3	37.3 ± 3.95	173 ± 6.14
Feb	11.2 ± 2.57	29.6 ± 8.25	26.8 ± 1.94
Mar	6.70 ± 0.81	13.5 ± 3.63	–
Apr	13.1 ± 0.73	12.1 ± 2.19	–
May	17.4 ± 2.45	16.5 ± 2.00	–
Jun	11.4 ± 1.44	9.39 ± 0.47	–
Jul	13.4 ± 2.11	12.5 ± 0.70	–
Aug	24.0 ± 4.58	15.4 ± 1.89	–
Sep	34.8 ± 5.23	33.2 ± 5.86	–
Oct	47.3 ± 7.93	33.7 ± 4.93	–
Nov	43.1 ± 6.15	157 ± 23.1	–
Dec	31.3 ± 7.40	304 ± 16.9	–
Year	281 ± 130	674 ± 57.6	–

Note that the average and one standard deviation for 2001–2018 were measured for each month of the year, and those of 2019 and 2020 indicate the eight results from a combination of input sources.

To comprehensively understand the CO_2_ emissions from the fires, we focus on the emissions in each state. The largest estimated monthly CO_2_ emissions were 181 ± 10.2 Tg CO_2_ month^−1^ in NSW, in December 2019 (Table 2). CO_2_ equating to 52% of the emissions from the fires in Australia was emitted from NSW across the 14 months evaluated in this study. The emissions from NSW in the latest year, March 2019 to February 2020, were 443 ± 40.4 Tg CO_2_ year^−1^, equivalent to 1.6 times average annual emissions through Australia in 2001–2018. In Victoria, 126 ± 8.50 Tg of CO_2_ was emitted over just two months between December 2019 and January 2020, and the annual emissions in the latest year, March 2019 to February 2020, were 149 ± 14.3 Tg CO_2_ year^−1^, equivalent to 53% of Australia’s average annual emissions for 2001–2018.

**Table 2:** Monthly time series of estimated CO_2_ emissions (Tg CO_2_ month^−1^) in six states and one territory between January 2019 and February 2020.

Year	Month	NSW	Queensland	SA	Tasmania	Victoria	WA	NT
2019	Jan	2.02 ± 0.40	5.31 ± 1.31	0.00 ± 0.00	18.9 ± 1.40	4.99 ± 0.75	5.79 ± 2.59	0.24 ± 0.12
	Feb	5.16 ± 0.88	7.03 ± 2.02	0.00 ± 0.00	2.77 ± 0.30	2.05 ± 0.37	12.5 ± 5.46	0.08 ± 0.01
	Mar	1.51 ± 0.18	1.09 ± 0.07	0.00 ± 0.00	0.38 ± 0.10	8.89 ± 3.98	1.52 ± 0.32	0.13 ± 0.03
	Apr	3.20 ± 0.83	0.84 ± 0.21	0.01 ± 0.01	1.81 ± 0.19	2.18 ± 0.44	3.55 ± 0.97	0.45 ± 0.13
	May	4.78 ± 0.80	0.98 ± 0.06	0.23 ± 0.14	1.40 ± 0.33	3.14 ± 0.80	4.40 ± 0.73	1.55 ± 0.23
	Jun	1.58 ± 0.16	1.52 ± 0.24	0.23 ± 0.10	0.24 ± 0.08	0.23 ± 0.13	0.96 ± 0.16	4.63 ± 0.63
	Jul	2.68 ± 0.30	2.77 ± 0.20	0.01 ± 0.01	0.00 ± 0.00	0.48 ± 0.12	1.16 ± 0.16	5.41 ± 0.47
	Aug	5.57 ± 0.57	4.79 ± 1.04	0.00 ± 0.00	0.12 ± 0.01	0.03 ± 0.03	0.4 ± 0.10	4.47 ± 0.56
	Sep	17.3 ± 3.19	11.4 ± 2.45	0.02 ± 0.02	0.41 ± 0.08	0.11 ± 0.06	0.74 ± 0.04	3.15 ± 0.31
	Oct	16.6 ± 2.46	8.96 ± 2.48	0.02 ± 0.02	0.33 ± 0.05	0.31 ± 0.05	1.5 ± 0.27	5.99 ± 0.28
	Nov	126 ± 15.6	16.0 ± 4.07	0.03 ± 0.01	0.58 ± 0.12	4.73 ± 0.06	7.88 ± 3.25	1.92 ± 0.19
	Dec	181 ± 10.2	23.7 ± 4.84	3.23 ± 0.38	1.93 ± 0.55	56.7 ± 2.33	33.9 ± 12.1	2.86 ± 0.19
2020	Jan	68.2 ± 4.90	4.23 ± 0.64	2.64 ± 0.30	4.09 ± 0.70	69.6 ± 6.17	22.0 ± 7.93	0.27 ± 0.04
	Feb	14.8 ± 1.16	0.98 ± 0.14	0.00 ± 0.00	0.01 ± 0.02	2.56 ± 0.12	6.18 ± 1.62	0.02 ± 0.01
Total		451 ± 23.2	89.6 ± 19.0	6.43 ± 0.55	33.0 ± 2.62	156 ± 11.3	102 ± 31.0	31.2 ± 2.54

The values show the average and one standard deviation of the eight results from a combination of input sources.

**Figure 2:**
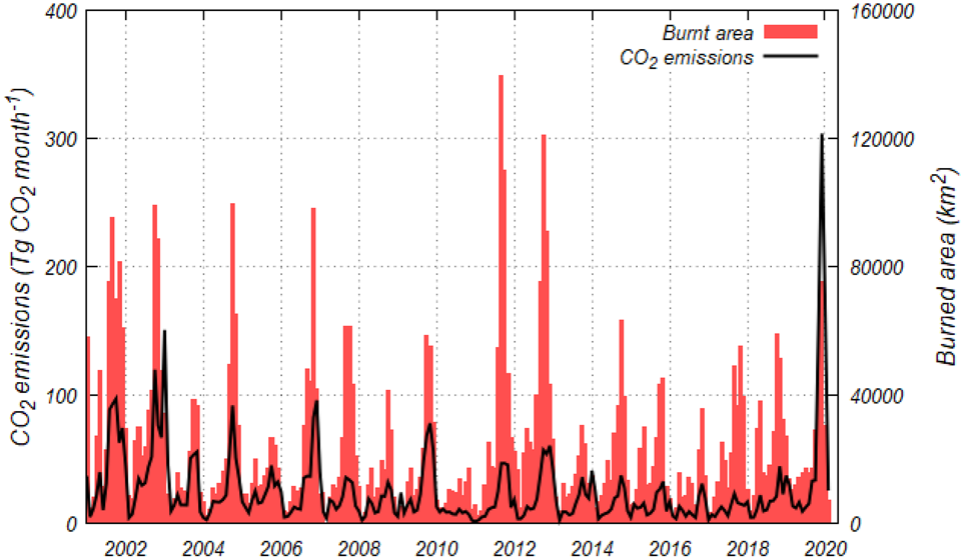
Monthly burnt area of LC–M and estimated CO_2_ emissions in Australia from January 2001 to February 2020.

**Figure 3:**
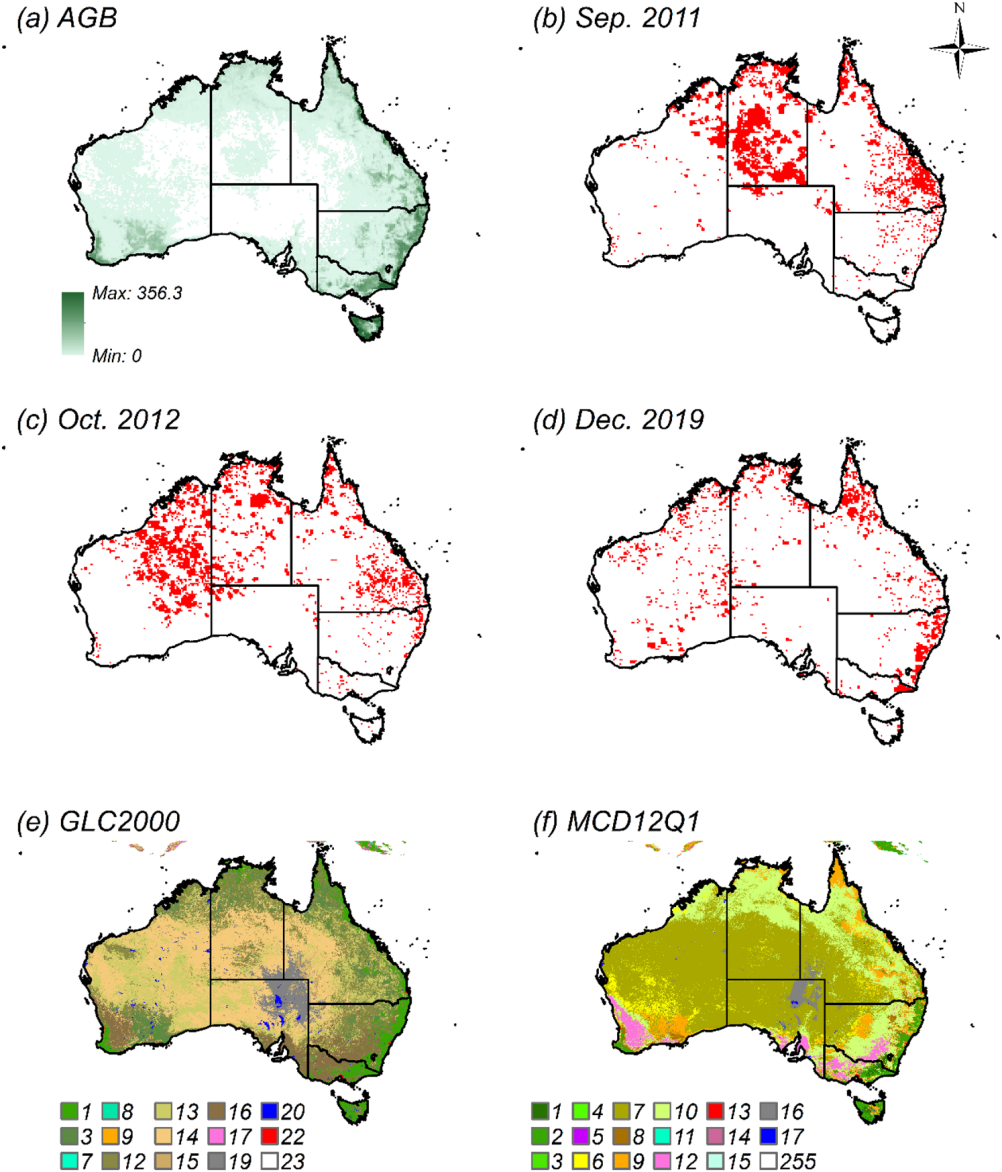
Distribution maps of AGB (Mg ha^−1^), burnt area, and LC. AGB map (**a**) was created by averaging the GEOCARBON and Globbiomass maps. Burnt area maps based on LC–M represent: (**b**) September 2011, (**c**) October 2012, and (**d**) December 2019. LC maps for 2018 at 500 m spatial resolution are: (**e**) GLC2000 and (**f**) MCD12Q1. The numbers in (**e**) and (**f**) indicate the category values for LC maps (Table S1). The grid size for maps from (**a**–**f**) was 20 km × 20 km. Maps were created with ArcGIS version 10.5 (https://www.arcgis.com/).

Although the highest monthly CO_2_ emissions through Australia since 2001 occurred in December 2019, the largest burnt area did not occur in this month (Fig. 2). The burnt areas in September 2011 and October 2012 were both larger than that of December 2019, however CO_2_ emissions were not as high during this month (46.8 ± 5.07 and 57.3 ± 7.58 Tg CO_2_ month^−1^, respectively). One of the reasons for this phenomenon is the above-ground biomass (AGB) density in the fire regions. We estimated the CO_2_ emissions from fires by multiplying the burnt areas by the AGB densities and few coefficients (Eqs. (1) and (2)). The inland areas in NT and WA, which comprised most of the burnt area in September 2011 and October 2012, respectively, had relatively low AGB distributions. Eastern NSW, however, which exhibited the highest emissions in December 2019, had a higher AGB distribution than the above two regions (Fig. 3).

**Figure 4:**
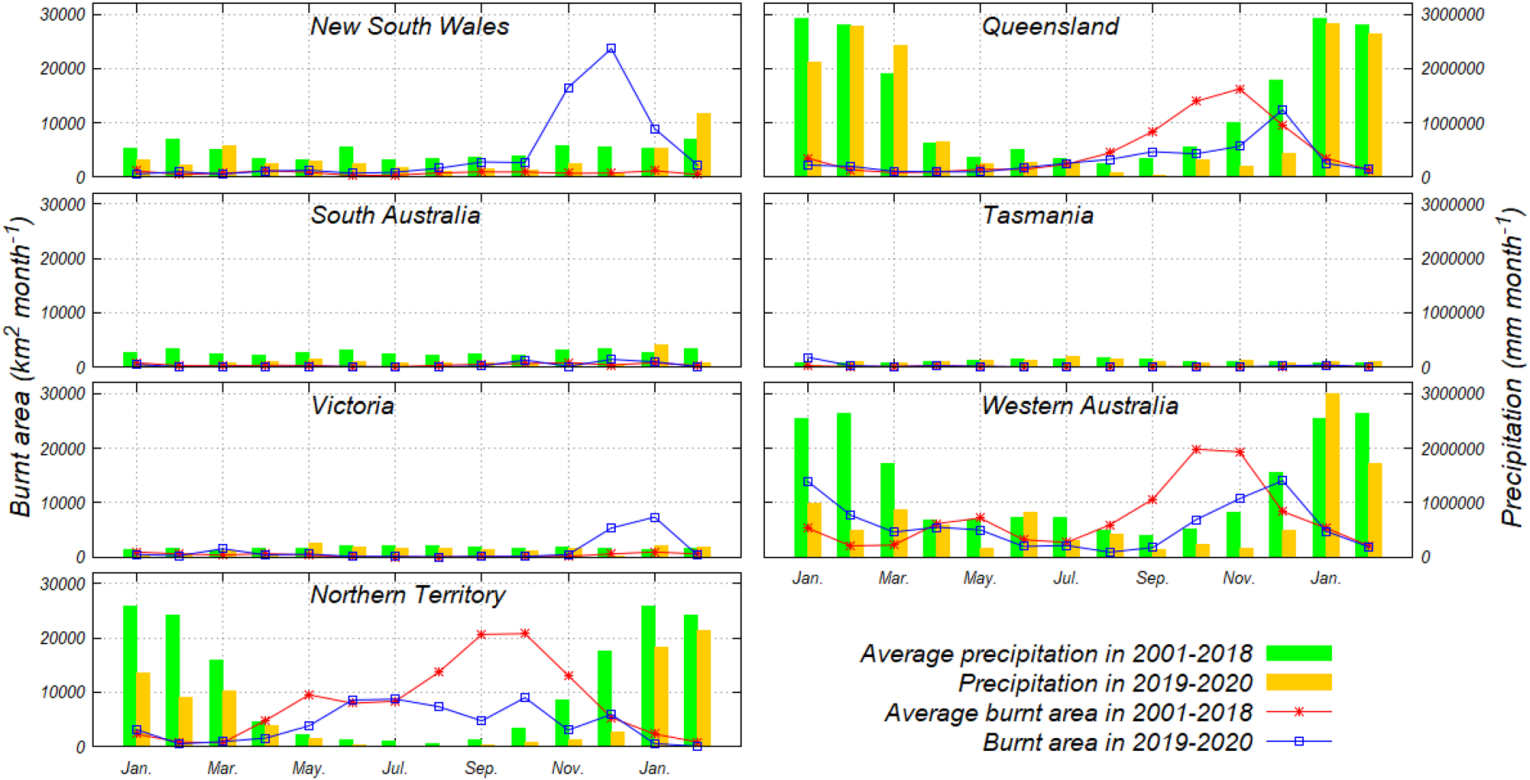
Comparison between monthly precipitation (mm) and monthly burnt area (km^2^) of LC–M in six states and one territory for 2001–2018, and for 2019–2020, respectively. Note that the horizontal axis shows the period from January to February of the subsequent year to avoid confusion from showing just two months for 2020.

The annual precipitation in 2019 was only 53% of the average for 2001–2018, with the monthly precipitation values in each region in 2019 often below the average precipitation of 2001–2018 (Fig. 4). The annual precipitation in 2019 in NSW, Queensland, and Victoria, the areas that emitted large amounts of CO_2_, were 50%, 73%, and 73%, respectively, of the average precipitation of each region for 2001–2018. The precipitation in NSW, Queensland, and Victoria for the three months from October to December 2019 was 29%, 29%, and 57%, respectively, of the average precipitation for the same months for 2001–2018. This was one month ahead of the period with significantly increased CO_2_ emissions (from November 2019 to January 2020). Focusing on the relationship between CO_2_ emissions, which was conducted using base 10 log transformation, and precipitation, all regions excluding SA had a negative correlation coefficient including Australia with the negative correlation (0.51) as shown in Figure S2. These results indicate that lower than average precipitation was one of the causes inducing the significantly greater CO_2_ emissions for the 2019–2020 seasons.

**Figure 5:**
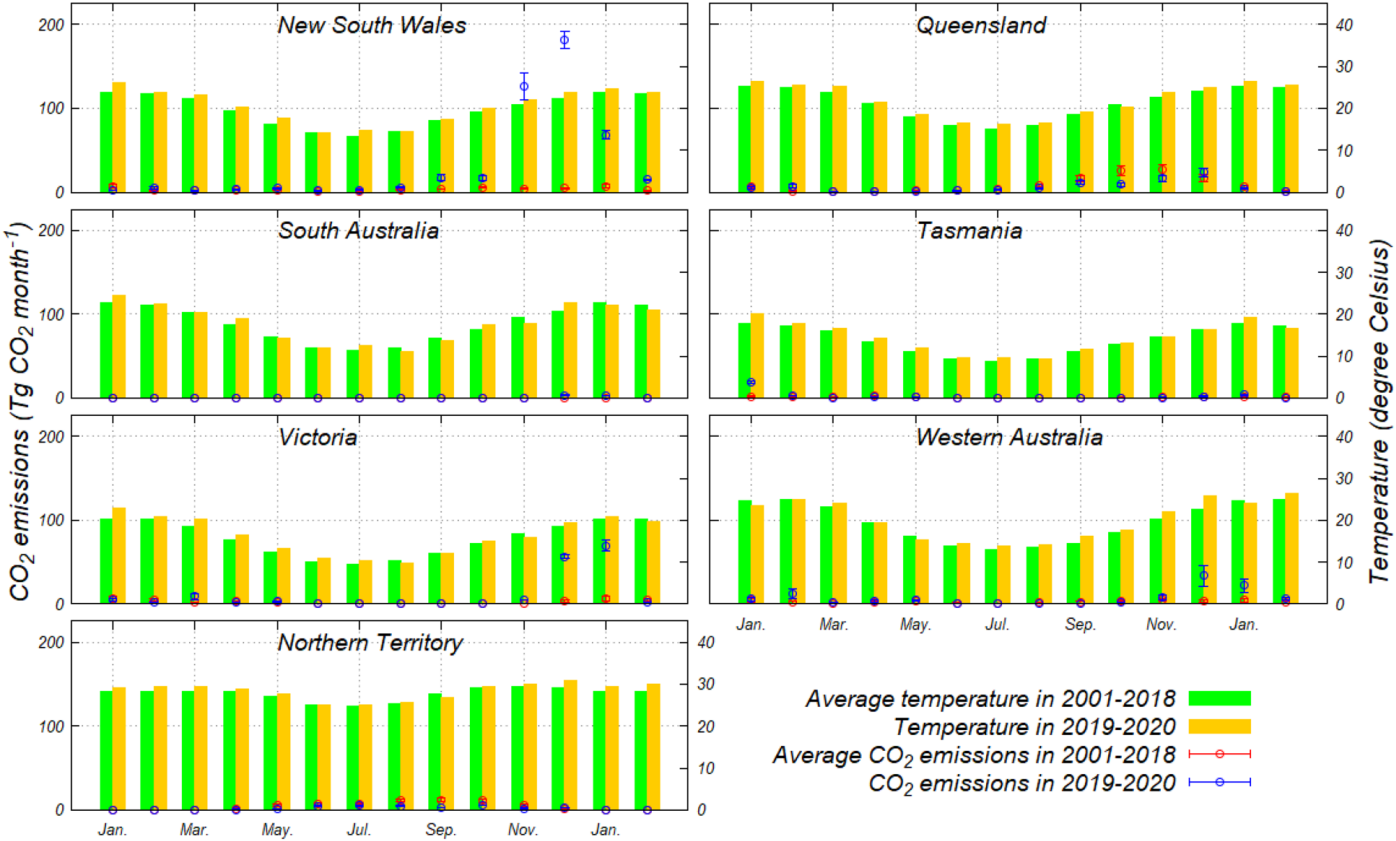
Comparison between monthly CO_2_ emissions (Tg CO_2_ month^−1^) and monthly mean temperatures (°C) in six states and one territory for 2001–2018 and for 2019–2020, respectively. Note that the average and one standard deviation of the CO_2_ emissions for 2001–2018 were measured for each month of the year, and those of 2019–2020 indicate the eight results from a combination of input sources.

Figure 5 shows the results of the monthly CO_2_ emissions and the monthly mean temperatures^14^ between the average values in 2001–2018 and the values in 2019–2020, respectively. The monthly mean temperatures for evaluation in each region (six states and one territory) were used in the capitals in each region as a representation. The temperatures in each region in 2019–2020 were on average 2–5% higher than those of 2001–2018. Although the CO_2_ emissions in NSW from November 2019 to January 2020 and in Victoria from December 2019 to January 2020 were conspicuously higher than those of 2001–2018, the significant difference does not prevail when compared with the other regions and the other periods. There was a weak correlation between the CO_2_ emissions and the temperatures in Tasmania, Victoria, and NT; however, no evident relationship was found in the other regions (Fig. S3). The higher-than-normal temperatures may have little effect on CO_2_ emissions directly; however, they indirectly contribute to the expansion of burning areas and CO_2_ emissions by causing the drying of fuel and soil.

## Discussion

### Effect of input data on the estimated CO_2_ emissions

We used land cover (LC) maps (GLC2000 and MCD12Q1), AGB maps (Globbiomass and GEOCARBON), and fire distribution (FD) maps, which are the map with high and nominal confidences (NC–M) and the map with high, nominal, and low confidences (LC–M) created from Moderate Resolution Imaging Spectroradiometer (MODIS) MOD14A1 product, to estimate the CO_2_ emissions from fires. We created the eight CO_2_ emission inventories in combinations (2^3^) using two LC maps, two AGB maps, and two FD maps. Note that the eight inventories were named by the three characters in each of the three inputs in the order of LCC (G: GLC2000 or M: MCD12Q1), AGB (W: GEOCARBON or E: Globbiomass), and FD (N: NC–M or L: LC–M) maps, for example, the GWN inventory is the combination of inputs in GLC2000 for LCC, GEOCARBON for AGB, and NC–M for FD (see Table S2 for every inventory). The highest CO_2_ emission inventory was MWL by 69.8 ± 92.6 Tg CO_2_ month^−1^, the smallest inventory was GEN by 55.7 ± 76.1 Tg CO_2_ month^−1^, and the difference was 25% (Fig. S4 and Table S2).

To understand the effect of AGB on the emissions, we compared the inventories with the same inputs of LC and FD (GWN and GEN, GWL and GEL, MWN and MEN, and MWL and MEL, respectively). The inventories using GEOCARBON had 11–14% more emissions than those of Globbiomass (Table S3). One of the reasons is the difference in AGB density between AGB maps. The AGB density in GEOCARBON is 14% higher in Australia than those of Globbiomass.

To understand the effect of LC on the emissions, we compared the inventories with the same inputs of AGB and FD (GWN and MWN, GWL and MWL, GEN and MEN, and GEL and MEL, respectively). The inventories using MCD12Q1 had 4–6% more emissions than those of GLC2000. CO_2_ emissions from forest areas with high AGB density were generally greater than the other LC areas. However, although the emissions of inventories using MCD12Q1 were greater than those of GLC2000, the forest areas in GLC2000 were 11% larger than MCD12Q1 (Table S4). The large difference region for the forest area is NT with low AGB density. Furthermore, NSW, Tasmania, and Victoria, where there is high AGB density, were evaluated to be 8–14% larger in forest area than GLC2000 on MCD12Q1. These results indicate the AGB density is more effective in CO_2_ emission estimation than LC.

To understand the effect of FD on the emissions, we compared the inventories with the same inputs of AGB and LC (GWN and GWL, GEN and GEL, MWN and MWL, and MEN and MEL, respectively). The inventories using LC–M had 6% larger emissions than those of NC–M. The method of creation of FD maps caused the difference. The LC–M was created from the three confidence flags on MOD14Q1 and includes the whole burnt area of the NC–M created from the two flags. We consider input sources to be of influence on the CO_2_ emission estimation, especially AGB density.

### Comparison with previous studies

Previous studies calculated burnt areas to cover 3.0 million hectares in the eastern states of Queensland and NSW^4^, 3.8 and 0.5 million hectares in the temperate forest of NSW and Victoria, respectively, for the fire season until 29–12–2019^2^, and 5.8 million hectares of temperate broadleaf forest across NSW and Victoria between September 2019 and early January 2020^5^ (Table S5). We measured the burnt areas from NC–M and LC–M to be approximately 4.3 and 4.5 million hectares, respectively, in NSW; 2.8 and 3.0 million hectares, respectively, in Queensland; and both 0.6 million hectares in Victoria between September and December 2019. Although the evaluated area and period do not completely match with the three previous studies, our results were 12–16% higher for NSW and 17% higher for Victoria than those of Nolan et al.^2^, 58–60% higher than those of Pickrell^4^, and 6–12% higher than those of Boer et al.^5^. The difference in the burnt areas between our results and the previous studies may be because we measured the burnt areas for the entire states, whereas there is the possibility that the previous studies concentrated on forest areas. MOD14A1 was mainly updated to decrease the omission errors in fire detection of all sizes and the obscuring fire detection by thick smoke. However, with MOD14A1, burnt areas were larger than actual owing to the low spatial resolution (1 km) because a burnt grid may have areas that are not burnt, smoldering areas, or flame areas. The factors that contributed to smaller evaluation for the burnt areas are the detection omission by burning periods outside satellite observation timing and the fire detection failure due to thick smoke or cloud cover as it is reported that the fire detection rate is 84% in Australia^15^. The CO_2_ emission estimation using a newly burned area product and the development of an accurate burned area product will be studied in future work.

**Figure 6:**
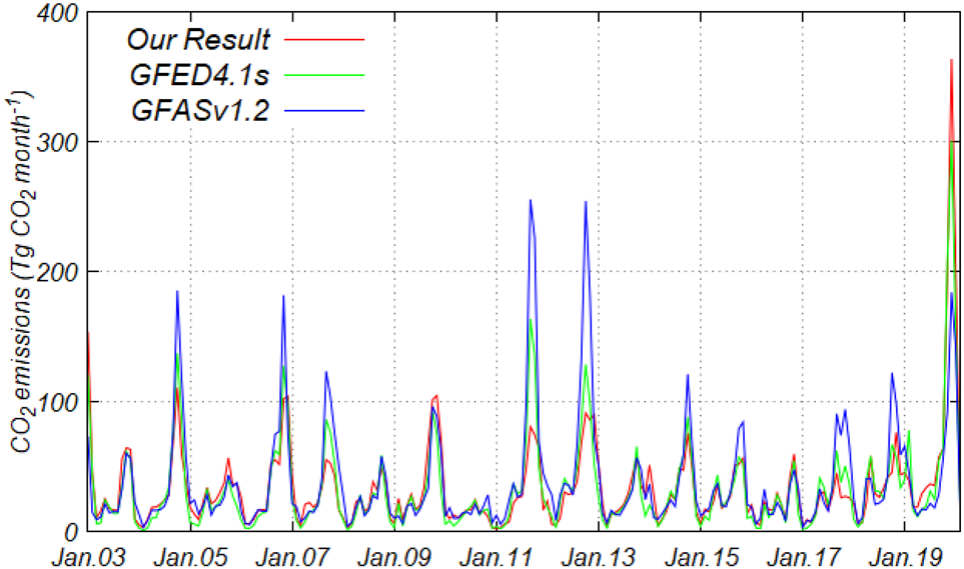
Comparison of monthly CO_2_ emissions from fires based on four inventories in AUST region (Fig. S5) from January 2003 to February 2020. The compared inventories are our results (red), GFED4.1 s (green), and GFASv1.2 (blue).

The combined CO_2_ emissions of Australia and New Zealand (AUST region in Fig. S5) were determined to be comparable with previous studies (Fig. 6). Data from the Global Fire Emissions Database (GFED4.1 s)^9^ and Global Fire Assimilation System (GFASv1.2)^7^ were used to estimate average monthly CO_2_ emissions from January 2003 to December 2019 to be 32.4 and 38.6 Tg CO_2_ month^−1^, respectively. Our estimated CO_2_ emissions (an average of one standard deviation for the eight results for combined input sources) in the same period were 33.5 ± 7.59 Tg CO_2_ month^−1^. These were 3% larger and 15% smaller than that of GFED4.1s and GFASv1.2, respectively. However, the uncertainty (one standard deviation) of our estimated emissions by input sources is 23%, and the average emissions in both of GFED4.1s and GFASv1.2 were within the uncertainty. One of the reasons for these products showing relatively close values against the different estimation approaches is that the product used a common input dataset. GFED4.1s uses GEOCARBON for adjusting the AGB as one of the input sources of the Carnegie–Ames–Stanford Approach model, which is the basis for calculating the carbon pools^9^. Furthermore, GFASv1.2 sets the several scaling parameters for the estimation to fit the emissions of GFED^9^.

### Uncertainty

The uncertainty in the estimated CO_2_ emissions was propagated from the remote sensing data, scaling coefficients, and features of this method itself. Regarding the uncertainty of remote sensing products, the overall fire detection rate of MOD14A1 has been calculated to be 84% for Australia^15^; the overall accuracies of GLC2000 and MCD12Q1 for LC maps were 68.6%^16^ and 73.6%^17^, respectively; and the root mean square error values of GEOCARBON^18^ and Globbiomass^19^ for the AGB maps were 87–98 Mg ha^−1^ and 52.8 Mg ha^−1^, respectively. The combination of these remote sensing datasets, which were used for the CO_2_ emissions estimation as inputs, introduced significant deviation into the estimation results. Our method used one-time fire instance for estimation and did not consider the burning term or the fire scale. Although the incinerated biomass density is considered in Eq. (2), biomass growth and recovery were not considered. These uncertainties influence each other and complicate evaluations of estimation results, which means that it is difficult to specify the uncertainty, similar to previous studies^9^.

Although we used BE and EF data sourced from Mieville et al.^20^ and Shi et al.^21^ as shown in Table S1, several authors have reported other values for Australia’s regions and LC categories. Regarding BE and EF for temperate forest, Paton–Walsh et al.^22^ reported the values of 0.88–0.91 and 1620 ± 160, respectively; furthermore, Guérette et al.^23^ reported 0.89–0.91 and 1620 ± 160 in NSW, 0.91–0.93 and 1650 ± 170 in Victoria, and 0.88 and 1621 ± 160 in Tasmania, respectively. Similarly, for savanna, Smith et al.^24^ reported the values of 0.90 ± 0.06 and 1674 ± 56, respectively, whereas Desservettaz et al.^25^ reported 0.90 ± 0.06 and 1536 ± 154, respectively. In forest, BE contributes particularly high levels of CO_2_ emissions and are 55–73% higher than the value we used. Therefore, to understand the impact of BE and EF on CO_2_ emission estimation as an examination, we estimated CO_2_ emissions using the BE and EF (g Kg^−1^) values of 0.895 and 1620 for forest, and 0.90 and 1613 for savanna, respectively, for January 2001 to February 2020 (Fig. S6). The estimated average monthly CO_2_ emissions was 40.0 ± 6.19 Tg CO_2_ month^−1^, which is 1.8 times higher than our result shown in Sect. 3. Both BE and EF are known to change, depending on the season and precipitation levels; they further strongly influence CO_2_ emission estimations from fires. Further verification of our results is required by comparing with the atmospheric concentration of CO_2_ using a top-down method, because the emissions estimated in this experiment were high compared to previous studies.

## Conclusions

This study presents the monthly changes in the time series and distribution of CO_2_ emissions from Australian fires across 2019–2020. In our results, although the burnt area was not the largest to have occurred since 2001, the CO_2_ emissions from this period were the highest, by 806 ± 69.7 Tg CO_2_ year^−1^ from March 2019 to February 2020. The emissions in the latest year were equivalent to 2.9 times the average annual emissions in 2001–2018, and 1.5 times total GHG emissions without land use, land use change and forestry emissions of CO_2_ equivalent for the whole of Australia in 2017. We found that lower than average precipitation and fires in high biomass density areas caused large CO_2_ emissions, and there was a correlation between CO_2_ emissions and precipitation for 2001–2020. The CO_2_ emission inventories shown in this study will be opened to include all inventories by combining them into an input dataset. The scope for future research in this topic includes a reflection of the time series change of biomass density and the incorporation of the scale and duration of fires into the estimation method to reduce the uncertainty associated with estimated CO_2_ emissions. Optimal BE and EF scenarios based on seasonal and precipitation changes, comparison of our estimated result with atmospheric concentrations, and the effect analysis of the emissions on regional/global carbon cycle need to be determined in future research. We expect that the CO_2_ emissions estimation and its evaluations from the catastrophic fires in Australia help to better assess the performance of climate and fire models.

## Materials and methods

### Estimation method of fire CO_2_ emissions

The remote sensing datasets were resampled at a 500 m spatial resolution, using the NEAREST function in ArcGIS version 10.5 to match the same spatial resolution. As MOD14A1 is a daily dataset, we created monthly burnt area datasets, including the number of fires occurring, to evaluate the burnt biomass in more detail.

CO_2_ emissions from fires (EMISSION, g CO_2_) were generally calculated using Eq. (1)^20,21,26^. However, this equation cannot evaluate the number of fires occurring within a single region over a period. Therefore, here we represented the decrease in biomass density by fires over a year using Eq. (2) to determine the AGB density in Eq. (1), though this method does not consider annual changes in biomass density.

1 $${\mathrm{EMISSION}}_{(m, p)}= {BA}_{(m, p)}\cdot {BD}_{(m, p)}\cdot {BE}_{(c)}\cdot {EF}_{(c)},$$

2 $${BD}_{(m, p)}={\sum }_{j=i+1}^{I}\left\{{Agb}_{(p)}\cdot {\left(1-{BE}_{(c)}\right)}^{j-1}\right\},$$

where *m* is the target month for calculating CO_2_ emissions, *p* is the grid position on the map, *c* is the LC categories of the grid (*p*), *i* and *I* are the cumulative number of fire occurrences until the last month (*m*–1) and the target month (*m*), respectively, BA is the burnt area (m^2^), BD is the total burnt biomass density (kg m^−2^), Agb is the biomass density (kg m^−2^) from the AGB map, BE is the burning efficiency (0 to 1), and EF is the emission factor of dry matter (g CO_2_ kg^−1^). We assigned the BE and EF values sourced from Mieville et al.^20^ and Shi et al.^21^ to fit the categories of GLC2000 and MCD12Q1, respectively, as shown in Table S1. Finally, the eight types of estimated CO_2_ emissions were combined into input datasets (2^3^), namely two FD maps (NC–M and LC–M), two LC maps (GLC2000 and MCD12Q1), and two AGB maps (GEOCARBON and Globbiomass), were applied as an ensemble average to estimate optimal CO_2_ emissions.

### Remote sensing data

The remote sensing products of FD, LC, and AGB were used to estimate CO_2_ emissions from fires.

FD maps were used with the Thermal Anomalies and Fire MODIS data product version 6 (MOD14A1), which provides daily fire data with 1 km spatial resolution^27,28^. Every fire pixel is assigned as having either low (0–30%), nominal (30–80%), or high (80–100%) confidence levels^29^. We used two types of FD maps with data on the number of fire occurrences, dependent on confidence level: NC–M, with high and nominal confidences; and LC–M, with high, nominal, and low confidences. We counted the number of fire occurrences recorded on the maps, and an ongoing fire on the same grid position in MOD14A1 daily datasets was considered a single fire.

LC maps, the Global Land Cover 2000 Project (GLC2000) data product^30,31^ and the MODIS Land Cover Type (MCD12Q1) Version 6 data product^17,32^, were used to obtain optimal scaling factors for each LC category. GLC2000 is a global LC map for the year 2000 and has 1 km spatial resolution. MCD12Q1 comprises a series of global LC maps from 2001 to 2018, with 500 m spatial resolution. The land use types used for the LC category were obtained from the Food and Agriculture Organization Land Cover Classification System (LCCS) for GLC2000 and from the International Geosphere-Biosphere Program for MCD12Q1. Note that MCD12Q1 of 2019 was applied to estimate the CO_2_ emissions for 2020, because the 2020 datasets were not published at the time of study.

AGB maps, namely the GEOCARBON global forest biomass map^18,33^ and the Globbiomass AGB map^34^, were used. The GEOCARBON map is a global AGB map with 1 km spatial resolution. Globbiomass is also a global AGB map with 25 m resolution; it is produced by the European Space Agency (ESA)^19^.

The Global Precipitation Measurement (GPM) level 3 product, with 0.1 degrees spatial resolution and monthly temporal resolution^35^, was used to evaluate the relationships between the estimated CO_2_ emissions, burnt areas, and precipitation. The monthly mean temperatures from ClimatView system^14^ from Japan Meteorological Agency were used to evaluate the relationship between CO_2_ emissions and temperatures.

## Supplementary Information

Supplementary Information
