# Supplementary material for: Estimation of carbon dioxide emissions from the megafires of Australia in 2019–2020
Source: Sci Rep. 2021 Apr 15;11:8267. doi: 10.1038/s41598-021-87721-x (PMC8050065; doi:10.1038/s41598-021-87721-x)
Supplement: Supplementary file 1 — Supplementary Information [file 41598_2021_87721_MOESM1_ESM.docx]

Supplementary Materials for

Estimation of carbon dioxide emissions from the megafires of Australia in 2019–2020

Tomohiro Shiraishi^*^, Ryuichi Hirata,

Correspondence to: shiraishi.tomohiro@nies.go.jp

**This file includes:**

Figures: S1 to S6

Tables: S1 to S5

References


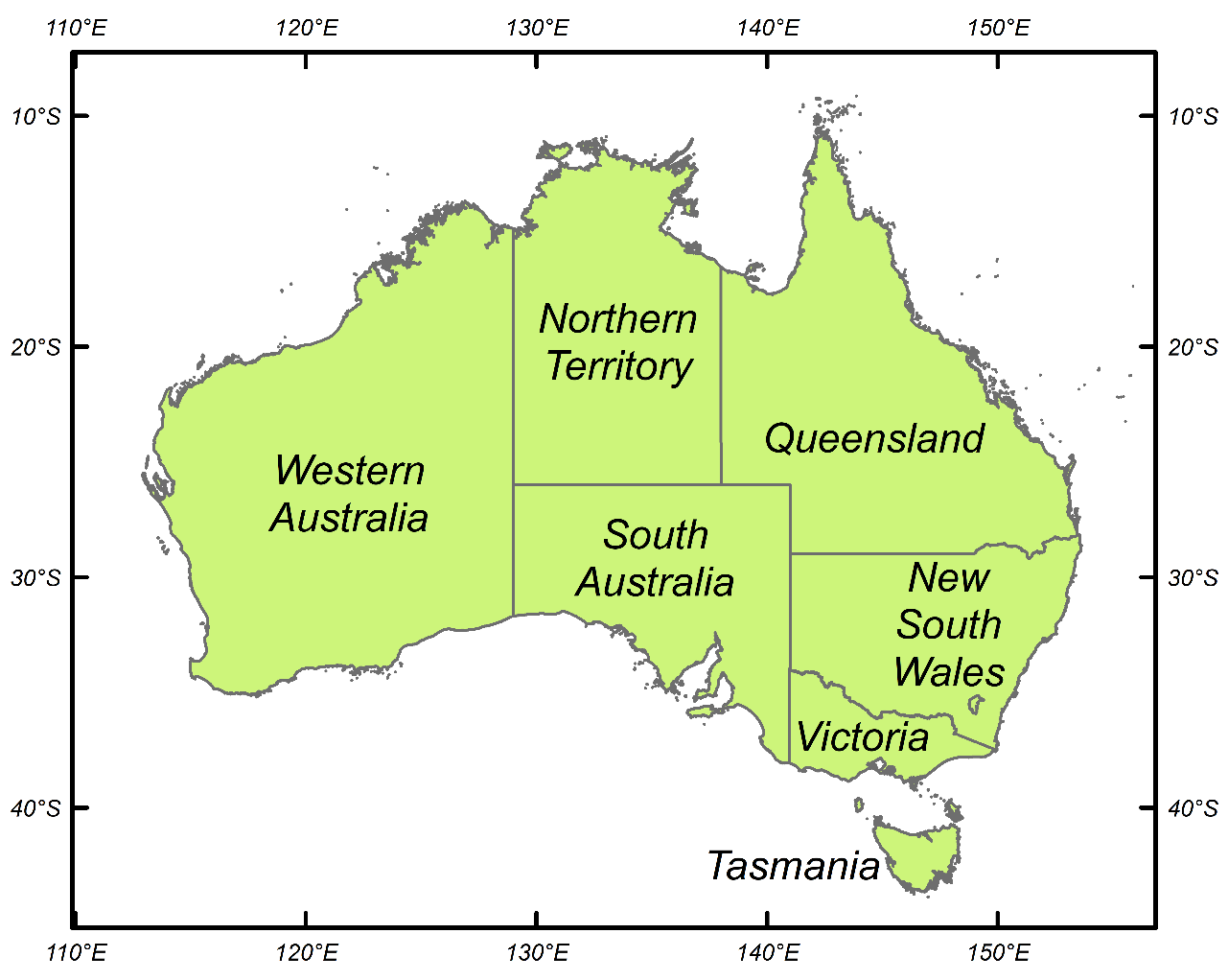


Figure S1. Study area including six states and one territory. Map was created with ArcGIS version 10.5 (https://www.arcgis.com/).


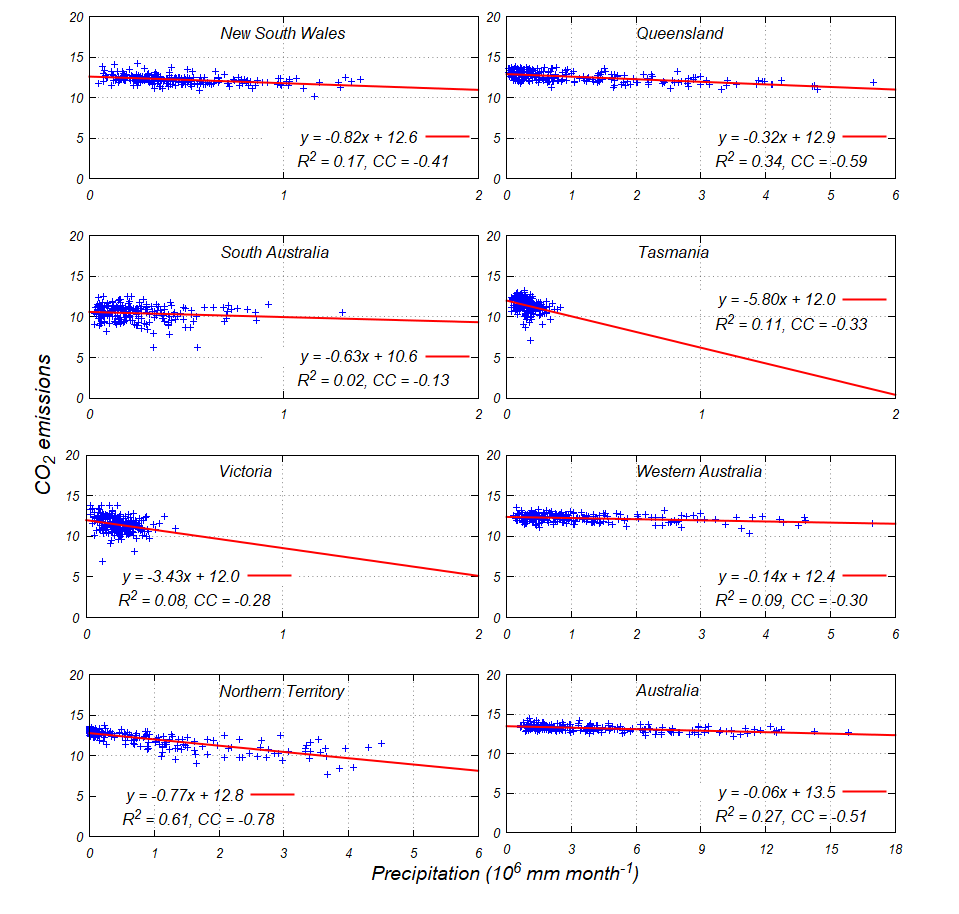


Figure S2. Correlations between CO_2_ emissions, which was conducted using base 10 log transformation, and precipitation in six Australian states, one territory, and the country as a whole. CO_2_ emissions from February 2001 to February 2020 were compared with precipitation from January 2001 to January 2020, (one month ahead of the estimated CO_2_ emission). R^2^ and CC are the coefficient of determination and the correlation coefficient, respectively.


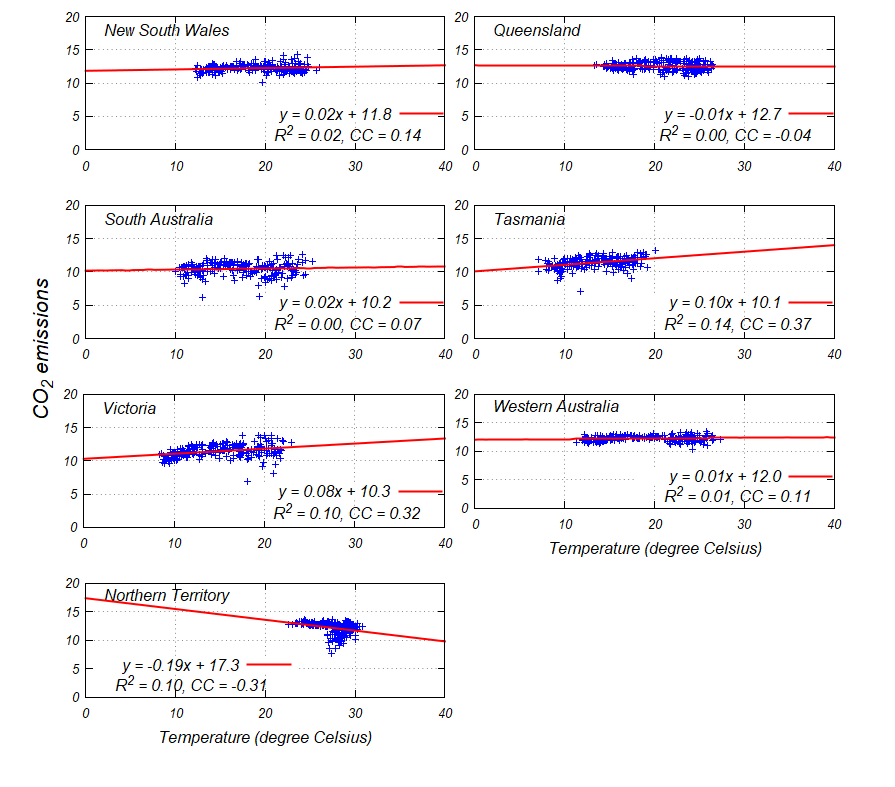


Figure S3. Correlations between CO_2_ emissions, which was conducted using base 10 log transformation, and monthly mean temperatures in six Australian states and one territory. R^2^ and CC are the coefficient of determination and the correlation coefficient, respectively.


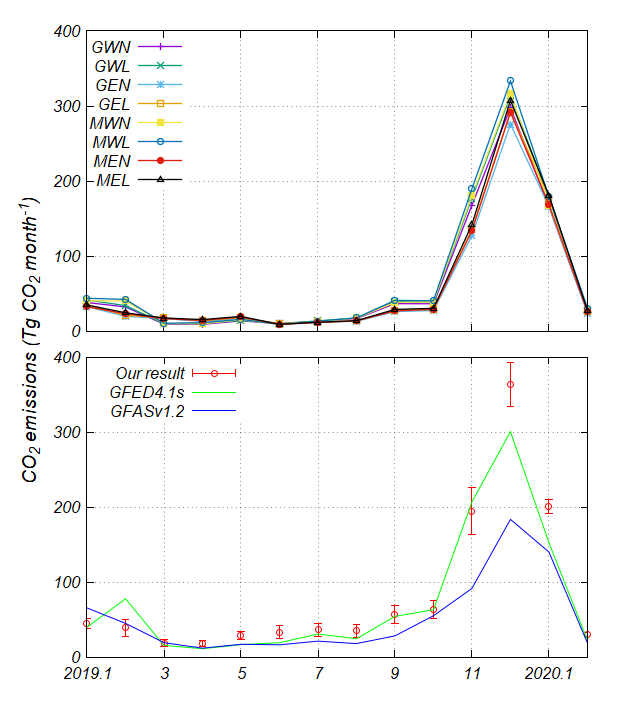


Figure S4. Monthly CO_2_ emissions for combination by input sources from January 2019 to February 2020. The top figure shows the emissions for every inventory in Australia, and the bottom figure is the average (with one standard deviation) emissions in AUST (Australia and New Zealand). The inventory names follow Table S2.


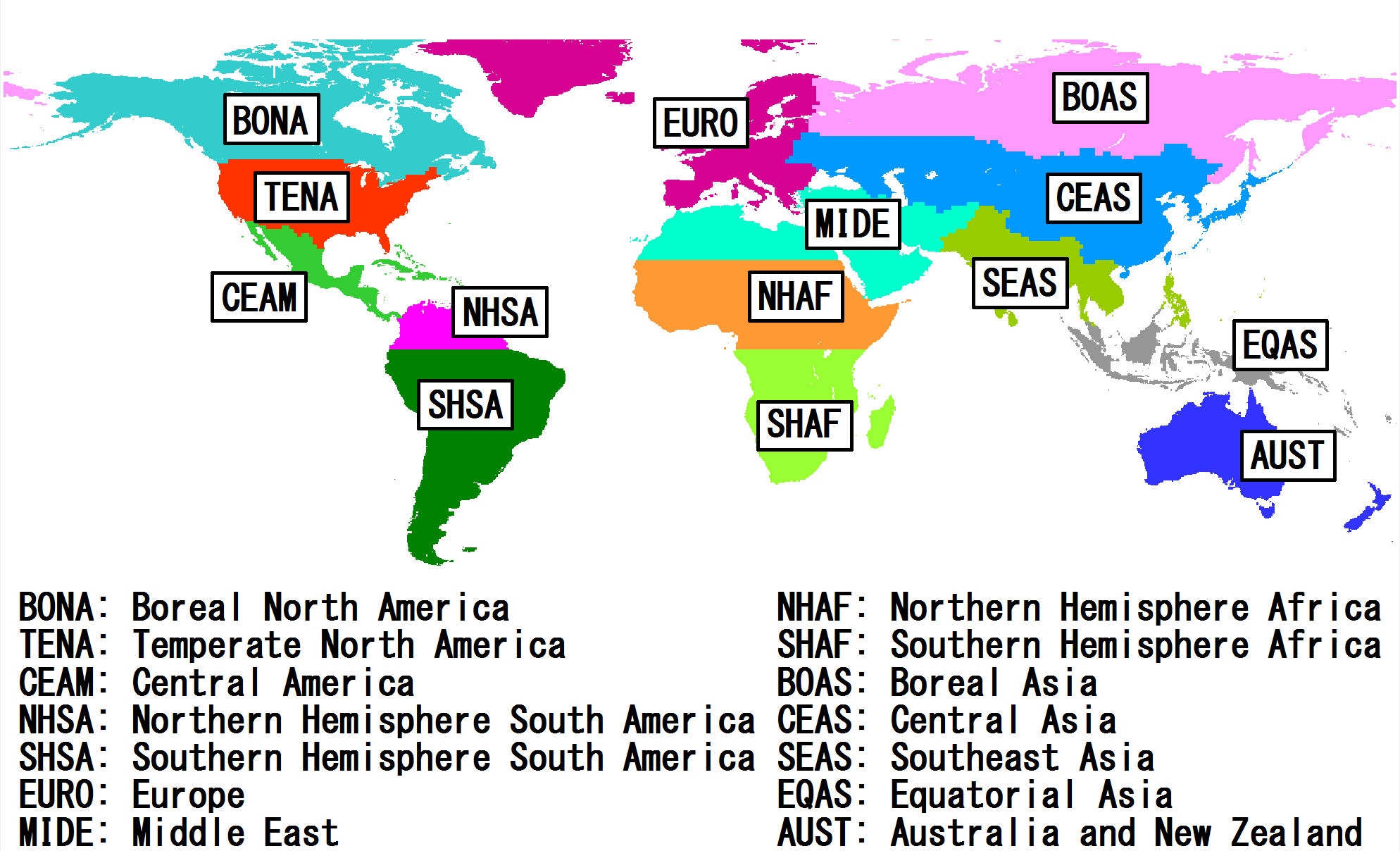


Figure S5. Spatial distribution map for evaluation, referred from van der Werf et al. (2010). Map was created with ArcGIS version 10.5 (https://www.arcgis.com/).


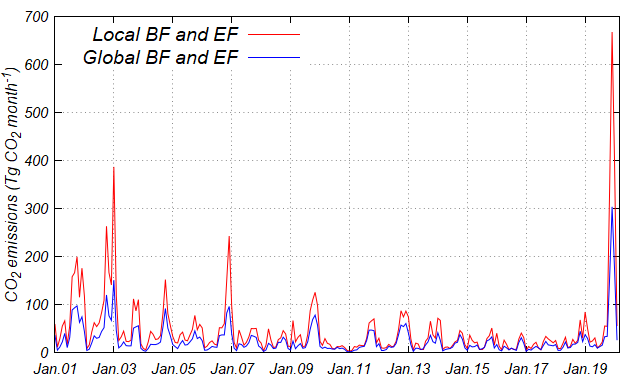


Figure S6. Comparison of estimated monthly CO_2_ emissions using local versus global BE and EF from January 2001 to February 2020. The estimated results using global BE and EF equate to our results in Fig. 6.

Table S1. Burning efficiency (BE) and emission factors (EF) for forest (F) and non-forest (NF) in GLC2000 and MCD12Q1 sourced from Mieville et al. (2010) and Shi et al. (2015). The characters in parentheses following NF category represent shrub (S), cropland (C) and others (O) classification categories for the evaluation.

| GLC2000 | | | MCD12Q1 | | | BE | EF (g CO_2_ kg^−1^) |
| --- | --- | --- | --- | --- | --- | --- | --- |
| No | F/NF | Category | No | F/NF | Category |  |  |
| 1 | F | Tree Cover, broadleaved, evergreen | 2 | F | Evergreen Broadleaf Forests | 0.25 | 1580 |
| 2 | F | Tree Cover, broadleaved, deciduous, closed | 4 | F | Deciduous Broadleaf Forests | 0.25 | 1569 |
| 3 | F | Tree Cover, broadleaved, deciduous, open | - | - | – | 0.4 | 1613 |
| 4 | F | Tree Cover, needle-leaved, evergreen | 1 | F | Evergreen Needleleaf Forests | 0.25 | 1569 |
| 5 | F | Tree Cover, needle-leaved, deciduous | 3 | F | Deciduous Needleleaf Forests | 0.25 | 1569 |
| 6 | F | Tree Cover, mixed leaf type | 5 | F | Mixed Forests | 0.25 | 1569 |
| 7 | NF(O) | Tree Cover, regularly flooded, fresh water | 11 | NF(O) | Permanent Wetlands | 0.0 | 0 |
| 8 | NF(O) | Tree Cover, regularly flooded, saline water | - | - | – | 0.0 | 0 |
| 9 | F | Mosaic: Tree Cover,  Other natural vegetation | 8 | F | Woody Savannas | 0.35 | 1591 |
|  |  |  | 9 | F | Savannas |  |  |
| 10 | NF(O) | Tree Cover, burned | - | - | – | 0.0 | 0 |
| 11 | NF(S) | Shrub Cover, closed-open, evergreen | 6 | NF(S) | Close Shrublands | 0.9 | 1613 |
|  |  |  | 7 | NF(S) | Open Shrublands |  |  |
| 12 | NF(S) | Shrub Cover, closed-open, deciduous | - | - | – | 0.4 | 1613 |
| 13 | NF(S) | Herbaceous Cover, closed-open | - | - | – | 0.9 | 1613 |
| 14 | NF(S) | Sparse herbaceous or sparse shrub cover | - | - | – | 0.6 | 1567 |
| 15 | NF(O) | Regularly flooded shrub and/or  herbaceous cover | - | - | – | 0.0 | 0 |
| 16 | NF(C) | Cultivated and managed areas | - | - | – | 0.6 | 1515 |
| 17 | NF(C) | Mosaic: Cropland, Tree Cover,  Other natural vegetation | 12 | NF(C) | Croplands | 0.8 | 1594 |
|  |  |  | 14 | NF(C) | Cropland,  Natural Vegetation Mosaics |  |  |
| 18 | NF(C) | Mosaic: Cropland, Shrub and/or grass cover | 10 | NF(C) | Grasslands | 0.75 | 1580 |
| 19 | NF(O) | Bare Areas | 16 | NF(O) | Barren | 0.0 | 0 |
| 20 | NF(O) | Water Bodies | 17 | NF(O) | Water Bodies | 0.0 | 0 |
| 21 | NF(O) | Snow and Ice | 15 | NF(O) | Permanent Snow and Ice | 0.0 | 0 |
| 22 | NF(O) | Artificial surfaces and associated areas | 13 | NF(O) | Urban and Built-up Lands | 0.0 | 0 |
| 23 | NF(O) | No data, Sea | 255 | NF(O) | Unclassified, Sea | 0.0 | 0 |

Table S2. Average and one standard deviation for the monthly CO_2_ emissions (Tg CO_2_ month^−1^) by the eight combinations of input sources in Australia from January 2019 to December 2020.

| LC map | AGB map | FD map | Inventory | Average | One standard deviation |
| --- | --- | --- | --- | --- | --- |
| GLC2000 | GEOCARBON | NC–M | GWN | 62.2 | 83.1 |
|  |  | LC–M | GWL | 66.1 | 87.9 |
|  | Globbiomass | NC–M | GEN | 55.7 | 76.1 |
|  |  | LC–M | GEL | 59.3 | 80.9 |
| MCD12Q1 | GEOCARBON | NC–M | MWN | 66.1 | 87.9 |
|  |  | LC–M | MWL | 69.8 | 92.6 |
|  | Globbiomass | NC–M | MEN | 58.2 | 79.7 |
|  |  | LC–M | MEL | 61.7 | 84.4 |

Table S3. Average above-ground biomass (AGB) densities (kg m^−2^) in forest and non-forest areas for two AGB products (GEOCARBON and Globbiomass).

| Region | GEOCARBON | Globbiomass |
| --- | --- | --- |
| New south wales | 4.03 | 3.97 |
| Queensland | 2.40 | 1.63 |
| South Australia | 0.17 | 0.32 |
| Tasmania | 22.2 | 19.1 |
| Victoria | 5.25 | 8.15 |
| Western Australia | 1.03 | 0.65 |
| Northern territory | 0.80 | 0.67 |
| Australia | 1.88 | 1.64 |

Table S4. Land cover areas (10^3^ km^2^) for three collected categories from two land cover maps (GLC2000 and MCD12Q1) in each region. The collected categories, which are “Forest” and “Non-forest” (Shrub and Cropland), are classified in Table S1.

| Region | GLC2000 | | | MCD12Q1 | | |
| --- | --- | --- | --- | --- | --- | --- |
|  | Forest | Shrub | Cropland | Forest | Shrub | Cropland |
| New south wales | 256.2 | 531.7 | 259.0 | 286.1 | 350.9 | 444.8 |
| Queensland | 399.0 | 1604 | 28.01 | 355.0 | 962.3 | 814.2 |
| South Australia | 17.42 | 797.7 | 140.7 | 31.00 | 916.8 | 196.6 |
| Tasmania | 85.96 | 7.528 | 7.248 | 93.54 | 0.312 | 8.597 |
| Victoria | 108.0 | 54.30 | 159.5 | 131.9 | 15.40 | 174.4 |
| Western Australia | 285.1 | 2705 | 200.3 | 225.1 | 2510 | 490.0 |
| Northern territory | 190.2 | 1368 | 36.67 | 75.94 | 1041 | 528.2 |
| Australia | 1344 | 7069 | 831.6 | 1201 | 5797 | 2658 |

Table S5. Comparison of the burnt areas with previous studies. NC–M and LC–M are the burnt area data we created from MOD14A1. The numbers in parentheses are the measured burnt area from September 2019 to January 2020. Note that the estimated burnt area by Pickrell (2019) was for the eastern states of Queensland and NSW, and that of Boer et al. (2020) was for temperate broadleaf forest across NSW and Victoria.

| Source | Period | Burnt area (million ha) | | | |
| --- | --- | --- | --- | --- | --- |
|  |  | NSW | Queensland | Victoria |  |
| NC–M | From September to December 2019  (From September 2019 to January 2020) | 4.3 (5.0) | 2.8 (3.1) | 0.6 (1.2) |  |
| LC–M |  | 4.5 (5.3) | 3.0 (3.3) | 0.6 (1.3) |  |
| Nolan et al. (2020)^2^ | fire season (until 29/12/2019) | 3.8 | - | over 0.5 |  |
| Pickrell (2019)^4^ | - | 3.0 | | | |
| Boer et al. (2020)^5^ | From September 2019 to January 2020 | 5.8 | | | |

**References**

Mieville, A. *et al.* Emissions of gases and particles from biomass burning during the 20th century using satellite data and an historical reconstruction. *Atmospheric Environment* **44,** 1469–1477 (2010).

Shi, Y., Matsunaga, T., Saito, M., Yamaguchi, Y. & Chen, X. Comparison of global inventories of CO2 emissions from biomass burning during 2002–2011 derived from multiple satellite products. *Environmental Pollution* **206,** 479–487 (2015).

van der Werf, G. R. *et al.* Global fire emissions and the contribution of deforestation, savanna, forest, agricultural, and peat fires (1997–2009). *Atmospheric Chemistry and Physics* **10,** 11707–11735 (2010).
